# Fire and Grazing Influences on Rates of Riparian Woody Plant Expansion along Grassland Streams

**DOI:** 10.1371/journal.pone.0106922

**Published:** 2014-09-05

**Authors:** Allison M. Veach, Walter K. Dodds, Adam Skibbe

**Affiliations:** 1 Division of Biology, Kansas State University, Manhattan, Kansas, United States of America; 2 Department of Geographical and Sustainability Sciences, University of Iowa, Iowa City, Iowa, United States of America; University of New South Wales, Australia

## Abstract

Grasslands are threatened globally due to the expansion of woody plants. The few remaining headwater streams within tallgrass prairies are becoming more like typical forested streams due to rapid conversion of riparian zones from grassy to wooded. Forestation can alter stream hydrology and biogeochemistry. We estimated the rate of riparian woody plant expansion within a 30 m buffer zone surrounding the stream bed across whole watersheds at Konza Prairie Biological Station over 25 years from aerial photographs. Watersheds varied with respect to experimentally-controlled fire and bison grazing. Fire frequency, presence or absence of grazing bison, and the historical presence of woody vegetation prior to the study time period (a proxy for proximity of propagule sources) were used as independent variables to predict the rate of riparian woody plant expansion between 1985 and 2010. Water yield was estimated across these years for a subset of watersheds. Riparian woody encroachment rates increased as burning became less frequent than every two years. However, a higher fire frequency (1–2 years) did not reverse riparian woody encroachment regardless of whether woody vegetation was present or not before burning regimes were initiated. Although riparian woody vegetation cover increased over time, annual total precipitation and average annual temperature were variable. So, water yield over 4 watersheds under differing burn frequencies was quite variable and with no statistically significant detected temporal trends. Overall, burning regimes with a frequency of every 1–2 years will slow the conversion of tallgrass prairie stream ecosystems to forested ones, yet over long time periods, riparian woody plant encroachment may not be prevented by fire alone, regardless of fire frequency.

## Introduction

Grasslands and wooded grasslands historically covered ∼30% of the world’s total land area, are responsible for ∼20% of global runoff, [1] and are threatened worldwide. Grasslands have become susceptible to woody plant encroachment within North America and across the globe [2]–[8]. Woody plant encroachment is occurring across numerous grassland ecosystems converting them into shrublands and forests. The timing required for this conversion may largely be attributed to interactions between climate, fire regime, herbivory, nitrogen deposition, and increases in CO_2_ concentrations [3], [9]–[11]. Conversion of grasslands to shrublands and forest may lead to shifts in terrestrial ecosystem functioning [6], [12], such as heightened carbon sequestration [13] and reductions in carbon mineralization [14]. Woody plant encroachment is thus leading to widespread ecosystem changes which may not easily be reversible [15].

Several factors may interact to influence the rate of woody plant expansion, thus the primary driver of woody encroachment is not easily discernible. However, fire frequency, as well as climatic or edaphic conditions within a region, may tightly control recruitment and subsequent expansion of woody plant species across grasslands [6]. Increases in woody shrub cover within watersheds at Konza Prairie (a tallgrass prairie ecosystem) are greatest with intermediate fire intervals of every 4 years [5], [16]. Annual fires may prevent additional recruitment of upland woody plant species, but cover may still increase, albeit much less than areas with a low burn frequency [16]. Other work in savannas indicates similar trends with high fire frequency reducing tree sapling recruitment and survival [17], [18]. Hence, high fire frequencies generally prevent further woody plant expansion within grassland ecosystems.

Other factors may greatly influence success of woody plant species growth and expansion, especially when coupled with fire interval. Large, ungulate herbivores in mesic grasslands (e.g., tallgrass prairie) may reduce the spatial extent of burning or fire intensity via grazing and removal of graminoid species [4]. Further, ungrazed watersheds with annual fires may not exhibit greater expansion of shrub cover, whereas grazed watersheds with annual fires have slight increases in expansion [19]. Alternatively, in savannas, grazing ungulates have been shown to reduce woody vegetation cover, potentially through selective grazing on woody seedlings [20], [21]. Thus, the effect of ungulate grazers on woody encroachment may depend on grazer resource preferences.

Terrestrial, grassland landscapes are globally subject to woody encroachment. However, North American tallgrass prairie streams are especially endangered because entire intact watersheds are even rarer than are remnant patches of prairie [22]. Small prairie streams have been characterized as open canopy systems with riparian zones dominated by grasses grading into riparian zones dominated by forests downstream [22]. However, riparian forests have begun to expand their native range within and outside of prairie riparian zones [2], [5]. Transitions from streams with open, grassy canopy to shaded, woody riparian areas could have consequences for stream hydrology and biogeochemistry causing potential ecosystem state changes to the streams themselves and downstream areas they drain to. Woody, riparian vegetation may reduce baseflow discharge rates and increase periods of no flow [23]. Woody plants access groundwater sources in riparian zones and can increase rates of evapotranspiration potentially causing declines in water yield [13], [24]. In addition, forested riparian zones intercept sunlight and shed leaf litter which increases terrestrial material input to streams potentially altering their trophic state [25] to an ecosystem reliant upon terrestrial carbon subsidies (i.e., strongly net heterotrophic) instead of one based on in-stream subsidies (net autotrophy). Such abrupt shifts in carbon subsidy source will likely alter resource availability for aquatic biota causing shifts in species assemblages [26]. Investigating woody encroachment in riparian zones is pressing as this phenomenon can greatly alter stream ecosystem function and structure.

In this study, we evaluated the magnitude and direction of riparian forest expansion across tallgrass prairie watersheds exposed to variation in grazing and fire frequency treatments. Water yield was also assessed for 4 watersheds to determine if any trends in the proportion of water import to export differed with temporal changes in riparian woody plant cover. We hypothesized that (*i*) across all watersheds, riparian woody cover would increase over time, (*ii*) watersheds exposed to grazing would exhibit greater increases in woody cover relative to those that are ungrazed due to low spatial extent of burning during controlled fires, (*iii*) watersheds exposed to a high fire frequency would exhibit little woody expansion relative to those with a 4 or 20 year fire frequency. Lastly, we hypothesized that (*iv*) water yield would significantly decrease over 25 years due to the increase in cover of deeper rooting woody plant species.

## Methods

### Study location

**Figure 1:**
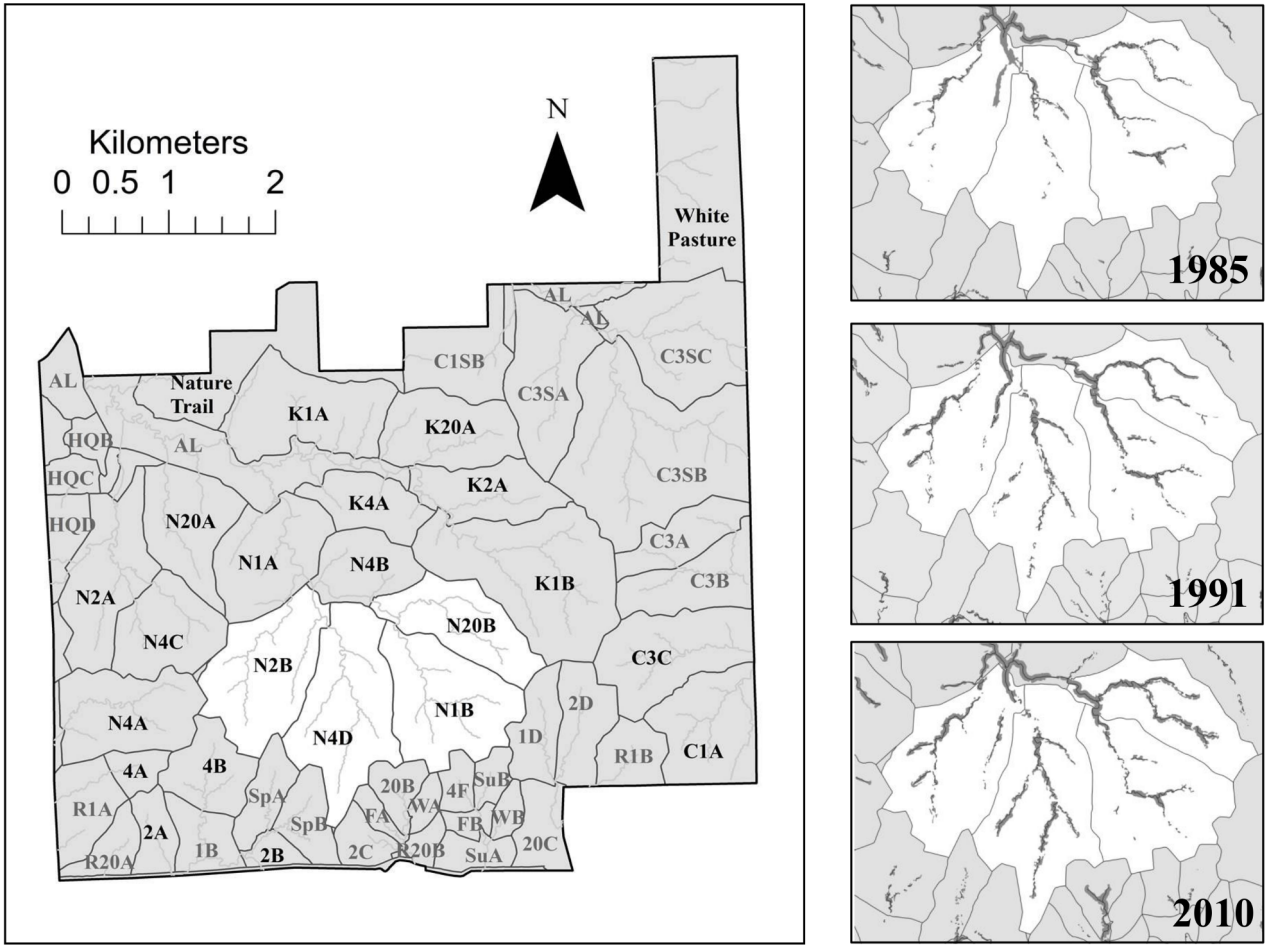
Spatial extent of woody plant species within a 30 m riparian buffer across the 4 watersheds of the Kings Creek basin monitored for stream discharge during 1985, 1991, and 2010. Woody vegetation cover within a 30 m buffer riparian zone is highlighted in gray for all three years for the 4 watersheds monitored. The 22 watersheds included in the analysis are labeled with black text whereas those not included are labeled in gray.

Konza Prairie Biological Station (KPBS) is a 3,487 hectare tallgrass prairie preserve and is part of the Flint Hills of northeastern Kansas. KPBS is privately-owned land by both the Natural Conservancy and Kansas State University. It is located ∼10 miles south of Manhattan, KS (KPBS Headquarters, 39°05′N, 96°35′W). KPBS granted an LTER permit (#200) for the work presented in this study. This study did not involve any protected or endangered species or involve collections of vertebrates. Any permission for research conducted at KPBS is approved through the Director of KPBS, John M. Briggs. Prescribed burning frequencies of variable intervals (every year, 2, 4, and 20 years) began in 1972. The site uses individual watersheds as experimental units under variable grazing and fire treatments. In 1987, 50 bison were introduced to a 469 ha portion of Konza Prairie and were allowed to increase through herd reproduction and other introductions until 1992 when the bison-grazed area expanded to an additional 480 ha encompassing 10 watershed units differing in their burn frequencies [27]. Watersheds are named by fire frequency (1, 2, 4, 20 years between burns), the inclusion or exclusion of native or cattle grazers (N or C), as well as specific drainage basin (K = Kings Creek north branch, S = Shane Creek) with the final letter assigned based on replicate number (A–D). For example, N04D is the fourth replicate (D) of a native grazed watershed (N) that is burned every 4 (04) years (Fig. 1, additional information regarding watershed treatments found at kpbs.konza.ksu.edu).

Konza Prairie receives, on average, slightly more than 800 mm of precipitation annually, and receives 75% of its precipitation in late spring and early summer with high interannual variability [28]. The site is characterized by limestone and shale bedrock with limestone forming benches and shales forming slopes resulting in a terrace-like landscape [29]. Across watersheds, upland vegetation is dominated by C_4_ grasses (e.g., *Andropogon gerardii, A. scoparius, Sorghastrum nutans*). In downstream riparian areas, oak gallery forest dominates (e.g., *Quercus macrocarpa, Q. muehlenbergii, Celtis occidenfalis, Ulmus americana*) [2]. In grazed, upland riparian zones where woody vegetation dominates, American elm (*U. americana*) and honey locust (*Gleditsia triacanthos*) are prominent, and in ungrazed headwaters woody riparian areas are dominated by bur oak (*Q. macrocarpa*), and chinkapin oak (*Q. muehlengbergii*) [30].

### Spatial analysis of riparian vegetation

Aerial images of Konza Prairie were taken during years 1985, 1991, and 2010. The 1985 images were originally flown to collect ∼1 m ground resolution data and were scanned to 200 dpi to avoid data loss. The 1985 aerial images were mosaicked and rectified using tools in ArcGIS (Version 10.1, ESRI 2012). The 1991 aerial consists of 1 m ground resolution, black and white imagery available as part of the USGS digital orthoquad (DOQ). The 2010 aerial is part of the 2010 USDA National Agricultural Inventory Program (NAIP) and is available in color at a resolution of 1 m (metadata for 1991 and 2010 images found at http://kansasgis.org/catalog/index.cfm).

Stream networks were created using digital elevation raster data and, using the Spatial Analyst expansion, riparian zones were defined around each stream. Wooded vegetation near the stream riparian corridor was digitized manually based on visual characterization of land cover and only vegetation within a 30 m buffer (30 m perpendicular to both sides of the stream) was analyzed across the 3 years. While the 30 m width is somewhat arbitrary, it is within the range commonly assumed to have the greatest success in stream conservation [31] and wider than many U.S. states define as protective of waters [32]. While some trees occur outside these widths, they are expected to only modestly impact the stream. We viewed the 2010 color image in black and white and found no discernible difference in our characterization of land cover. The percentage of wooded vegetation (trees and shrubs) within buffers was determined and standardized by stream length within a watershed. Due to changes in fire frequency and other management treatments over time or the confounding effect of multiple wild fires and partially burned watersheds, only data for 22 out of 54 watersheds were retained for further analyses. Of these 22 watersheds, half were grazed, but only 2 were grazed by cattle so no differences between native and cattle grazed watersheds were determined in this study.

A linear regression model was performed for each watershed separately using year (1985, 1991, and 2010) as the independent variable and percentage of wooded vegetation within the buffer as the dependent variable. The regression slope estimate was then used to represent the rate of wooded vegetation increase from 1985–2010. All slopes were used regardless of their statistical significance because we were interested in the direction and rate of change or lack thereof. Only using significant slopes would bias toward watersheds with large amounts of change and against watersheds where no change was evident.

Using the non-parametric, 2 dimensional Kolmogorov-Smironov test [33], we found a potentially non-linear (bi-variate) response (p<0.05) of woody vegetation encroachment to burning frequency. Therefore, a multiple, linear regression model and a segmented (breakpoint) regression model were applied to determine what factors influence the rate of expansion or contraction of riparian wooded vegetation. Normal probability plots and quantile-quantile plot of slope residuals confirmed that data did not violate any assumptions regarding normality. The cumulative number of burns that had taken place between 1985 and 2010 for each watershed was collected through the Konza Prairie Biological Station LTER network burn history database [34].

The presence of grazers and whether riparian wooded vegetation was present prior to Konza Prairie were also used as predictor variables. Lastly, as a surrogate for proximity of propagule sources, a 1939 aerial image was used to visually distinguish the historic presence or absence of woody vegetation along streams within each watershed. This image was created in the same manner as the 1985 image. While the image quality was poorer than the more recent images used, the image did allow for determination of areas with high densities of large trees. Since the presence or absence of trees in the riparian zone of each watershed was a categorical variable, high precision in cover was not necessary for this analysis. This approach was taken because preliminary examination revealed some areas were largely void of woody vegetation (e.g., the southernmost watersheds), but other riparian corridors (primarily those in the northwest corner of current Konza Prairie) were already wooded before the site was established.

### Water yield

If riparian wooded vegetation is increasing over time, water yield could also change temporally. Annual water yield was calculated for each of the 4 watersheds (N01B, N02B, N04D, N20B) that have long-term continuous discharge data spanning 1987 to 2010 (LTER dataset codes ASD02, ASD04, ASD05, ASD06) [35]. Discharge was measured over 5 minute periods using Druck pressure transducers at v-notch weirs. All of these watersheds impacted by bison, but have varying fire frequencies. Precipitation and air temperature were collected from the LTER Climate and Hydrology Database Projects database [36]. Discharge data were missing after 2006 for watershed N04D due to pressure transducer malfunctioning. Precipitation and mean discharge were summed per year to calculate annual water import and export, respectively, for each watershed. Water yield was then calculated as the proportion of discharge to precipitation standardized by watershed size. Summing these values on an annual basis prevented any temporal autocorrelation for each watershed as indicated by a correlogram.

A multiple, linear regression model was performed for each watershed separately to determine if water yield changed over time and with average, annual air temperature. Finally, a one-way ANOVA was performed to determine if water yield differed among watersheds. A Tukey’s HSD test in conjunction with Bonferroni corrections was then used to determine watershed specific differences in water yield. All regression analyses and ANOVAs were performed in the R programming language using the *segmented*, and *stats* packages (Version 2.13.1, R Development Core Team 2013). Temporal autocorrelation for water yield estimates was tested via correlograms computed in the *stats* package as well (Version 2.13.1, R Development Core Team 2013) whereas Kolmogorov-Smironov tests were performed using Statistica (Version 10.0, Statsoft, Tula, OK).

## Results

### Riparian vegetation spatial analysis

Analyses of 30 m riparian buffers revealed an increase in wooded vegetation over time among all watersheds except one (Watershed 2B, β = −0.06). Except for this watershed, all exhibited a positive (although not necessarily significant) rate of woody expansion, regardless of fire frequency or historical presence of woody vegetation (Fig. 1).

**Figure 2:**
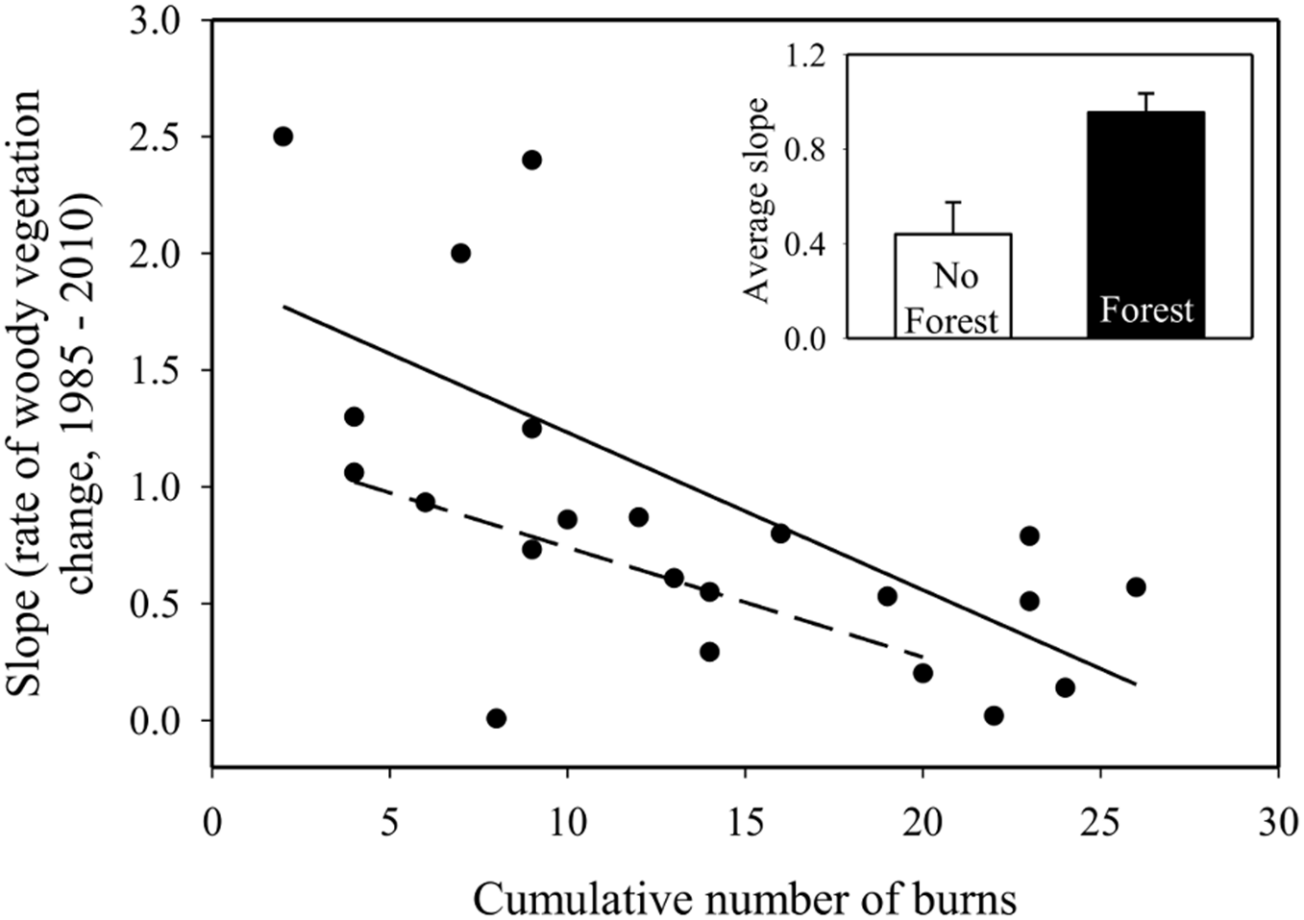
The association between the linear regression slopes calculated for each watershed’s change in riparian vegetation from 1985–2010 and the cumulative number of burns since 1980 using a multiple, linear regression model. Separate regression lines are present for watersheds without riparian woody vegetation present (open circles, dotted line), and watersheds with riparian, woody vegetation present historically (closed circles, bold line). The average slope of watersheds (rate of woody riparian expansion) with riparian forest was greater than those without forest historically (upper right panel).

Linear regression models indicated that the cumulative number of burns between 1980 and 2010, and the historical presence of woody vegetation, significantly predicted the rate of riparian vegetation expansion (P<0.01, Adj. R^2^ = 0.45, F_3,18_ = 6.61; Fig. 2). Further, the average rate of expansion of watersheds with forest present historically was significantly greater than those without forest (P = 0.05, T = −2.09, df = 20; Fig. 2). In other words, watersheds with trees present in the 1930s exhibited more rapid expansion of woody riparian vegetation. The presence of grazers did not influence expansion rates. Since the presence or absence of bison grazers across watersheds did not affect expansion rates, we also separated out watersheds with bison introduced in 1987 and 1991 and still found that grazers did not influence rates of riparian, woody expansion regardless of timing of their introduction.

**Figure 3:**
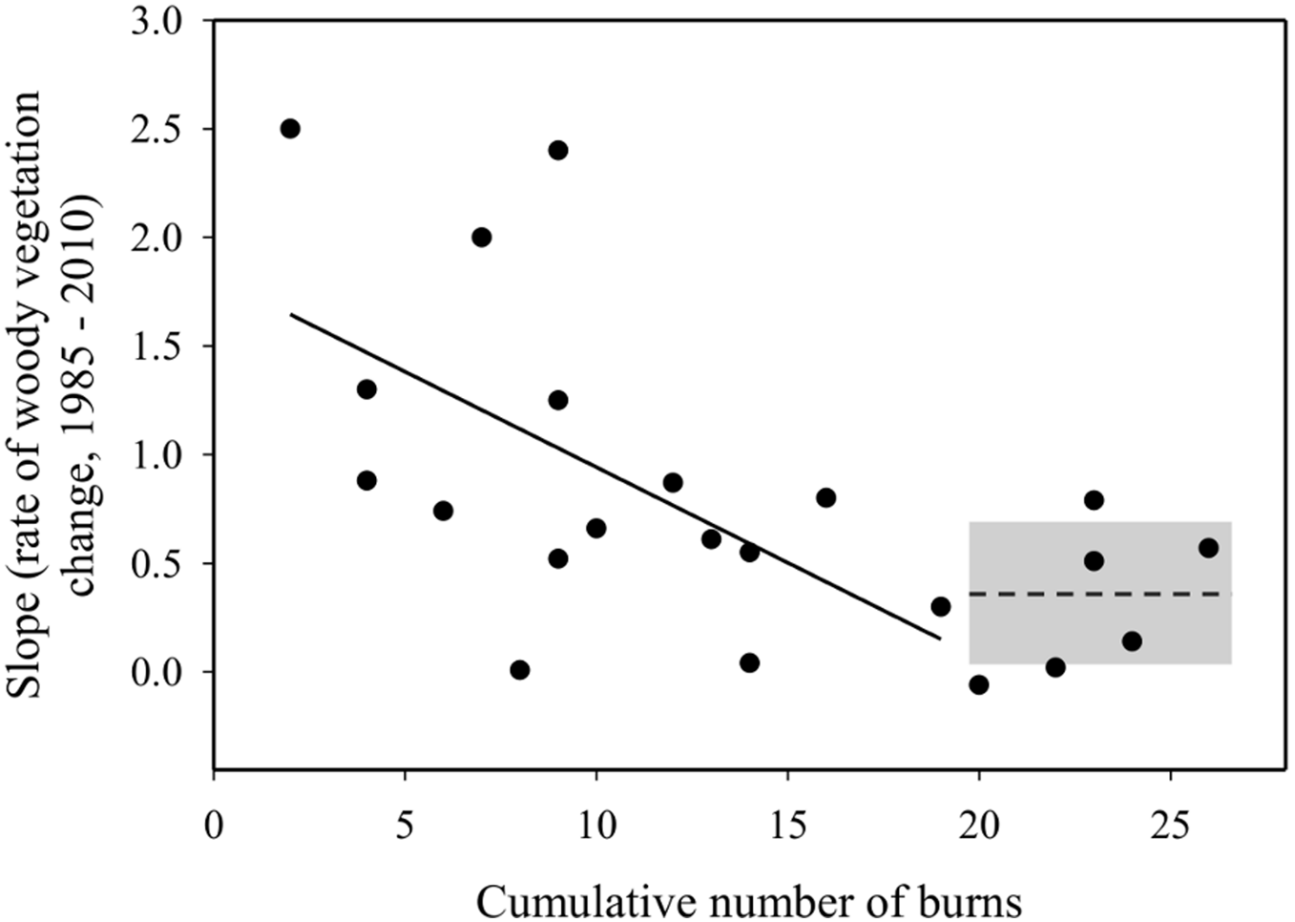
The association between the linear regression slopes calculated for each watershed’s change in riparian vegetation from 1985–2010 and the cumulative number of burns since 1980 using a segmented regression model. A breakpoint was detected at ∼19 burns. The bold line represents the linear regression line for the significant portion of the regression model (watersheds with burns <19 over the 25 year record). A dashed line represents the mean of the slope of watersheds with cumulative burn >19. Gray box represents 95% confidence bands about the mean value for watersheds burned more frequently than every 1.6 years.

A breakpoint was detected between burn frequency and woody expansion rate at 19 (±3.5 S.E) burns over the 25 years or at about 1.6 years between burns (Overall model: Adj. R^2^ = 0.32, Fig. 3). Only the regression model fit on the side of the breakpoint with fewer than 19 burns had a significant slope, indicating that the cumulative number of burns significantly predicts the rate of woody expansion (P = 0.02, β = −0.09, Adj. R^2^ = 0.2; Fig. 3). Due to the low number of watersheds which had >19 burns over the study period, the segmented regression did not indicate a significant regression slope after the break. Thus, we elected to use a one-sided Student’s t-test to test the hypothesis that woody vegetation increases at the greatest burn frequencies. The rate of woody expansion for watersheds with a cumulative number of burns greater than 19 were marginally significant from zero (Mean = 0.33, P = 0.07, T = 2.35, df = 5; Fig. 3) indicating that burning regimes implemented more frequently than every 1.6 years may not necessarily prevent woody encroachment.

### Climatic variables and water yield

Annual precipitation ranged between 503 to 1115 mm across the study period and average annual temperature ranged between 11.4 to 14.8°C. Both were highly variable over time.

**Figure 4:**
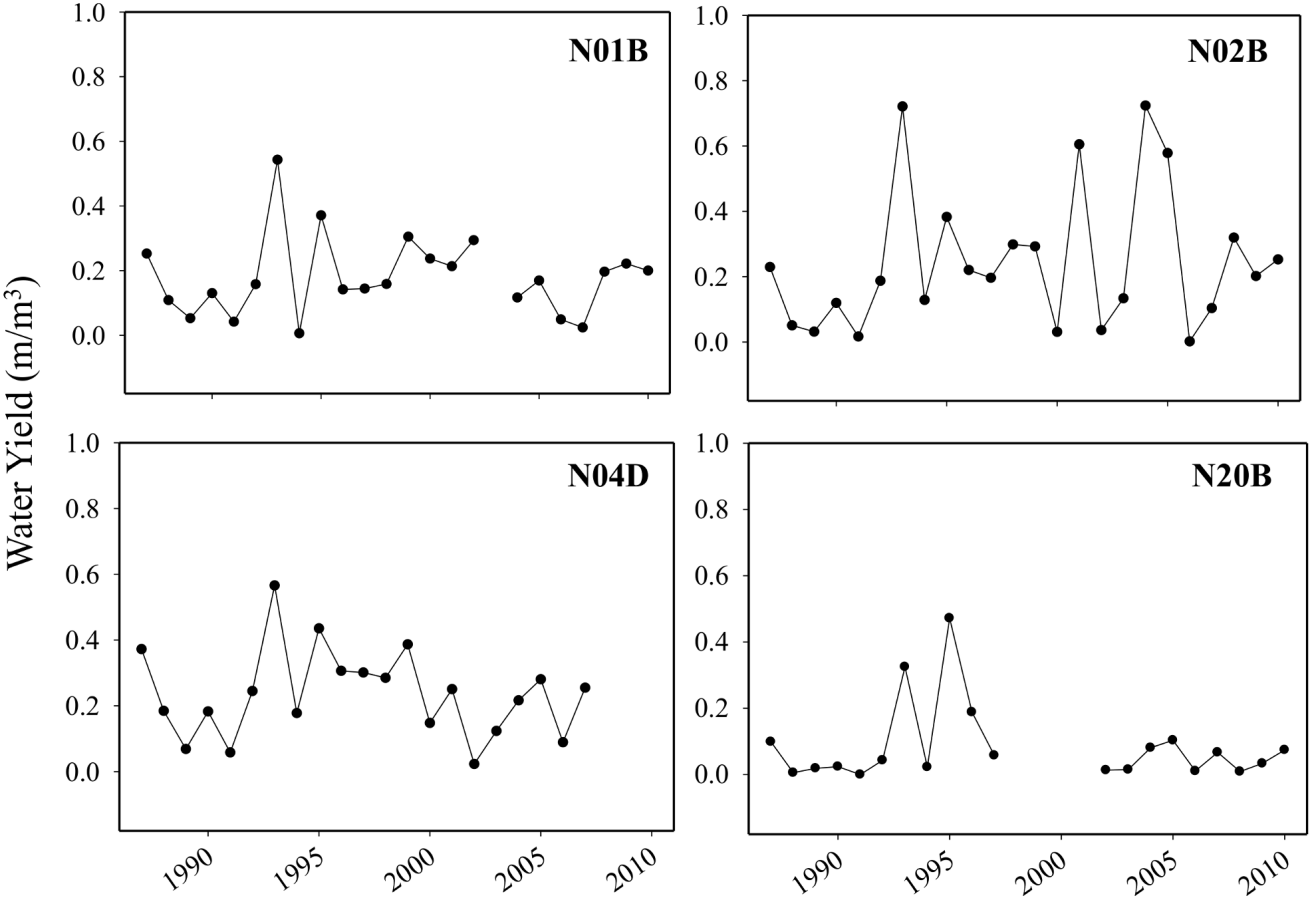
The ratio of discharge to precipitation standardized by watershed size (water yield) for a 1 year (N01B), 2 year (N02B), 4 year (N04D), and a 20 year (N20B) burned watershed. No watersheds exhibited a general trend in water yield over time, but N02B and N04D water yields were statistically higher from N20B (P<0.01).

Annual water yield across the four gauged watersheds never exceeded 0.72 m precipitation/mm runoff, and on average was 0.19 indicating overall only ∼1/5 of precipitation was exported as stream runoff. Linear regression models indicated that none of the watersheds water yields differed over time or with temperature (P≥0.28 across all 4 models). However, N02B and N04D had greater water yield on average than N20B (P<0.01; Fig. 4, Table 1).

**Table 1:** Characteristics of the four study watersheds.

					*% woody vegetation in buffer*			
Watershed	Area (ha)	Total Burn #	Slope	Total StreamLength (m)	1985	1991	2010	Average Water Yield
N01B	120.7	23	0.51	3937	40.2	56.9	57	0.18 (0.03)
N02B	77.6	12	0.87	2387	43.6	63.5	69.6	0.25 (0.05)
N04D	125.6	7	2	3886	22.3	50.9	76.3	0.24 (0.03)
N20B	84.4	4	1.3	2327	52	78.5	89.7	0.09 (0.03)

Total burn # refers to the cumulative number of burns between 1985 and 2010.

## Discussion

### Factors influencing riparian, woody vegetation expansion

Nearly all watersheds within this study have experienced riparian woody vegetation expansion since the establishment of Konza Prairie (Fig. 1). None of these studies have focused explicitly on riparian cover, but rather on total cover, and have provided similar data showing that across Konza Prairie, forested land has increased from 5 ha in 1859 to 274 ha in 2002 (72% areal increase) [5], [24]. The rate of riparian woody vegetation expansion was significantly predicted by the cumulative number of burns taken place between 1985 and 2010. High fire frequency can reduce woody vegetation cover in some grassland ecosystems, although none of these studies have focused on riparian vegetation. For example, previous studies found that cover and density of both shrubs and trees at Kruger National Park declined by 40 years partially due to frequent, prescribed fires [37]. Moreover, woody cover declined 40–50% after 2 annual burns relative to unburned plots in the South Texas Plains [38]. Within tallgrass prairies, annual fires have prevented woody vegetation expansion, whereas watersheds subjected to intermediate fire frequencies (every 4 years) have had substantially greater tree and shrub density [4].

Our study indicates that the rate of riparian woody vegetation expansion is lessened with greater fire frequency, but even in annually burned watersheds, fires cannot prevent some encroachment of woody vegetation within riparian corridors (Fig. 2, 3). Segmented regression results suggests that a threshold may be reached for woody vegetation cover at ∼19 burns over the 25 year study period signifying that there is a change in the way riparian woody vegetation cover responds to fire when implemented every 1–2 years (Fig. 3). Shrub species can persist and still increase in cover even in frequently burned areas [5], and apparently the tree-dominated riparian zones can also persist in the face of fire. *Cornus drummondii*, a common, clonal shrub species at Konza Prairie, forms “islands” which exclude grassy species [39], but may contain other woody species, such as tree seedlings, therefore promoting the expansion of forest [40]. Further, fire may cause short-term periods of high resource availability, causing enhanced recovery and growth of *C. drummondii* [41] and similar effects may occur with respect to riparian trees.

The presence or absence of woody riparian vegetation prior to the beginning of Konza Prairie significantly predicted its rate of expansion (Fig. 2). Historical records suggest that tree cover was common along the nearby Kansas River (about 5 km from Konza Prairie). The earliest written records from the Fremont expedition in 1843 taken along the Kansas River upstream of present-day Topeka note, “We halted for dinner, after a march of about thirteen miles, on the banks of one of the many little tributaries to the Kansas, which look like trenches in the prairie, and are usually well timbered.” [42]. The railroad surveys of Konza Prairie from the 1850’s indicate little tree cover on site. Hence, propagule sources have been close to Konza Prairie for at least a hundred years [43], but there was little woody vegetation on site in both the 1850’s and the 1930s. Watersheds which had forest along the riparian area in 1939 had a significantly higher rate of increase for vegetation cover relative to watersheds without forest. While presence of trees in the 1930s is a weak surrogate for proximity of propagules, this result could indicate propagule limitation for woody recruitment and expansion. However, we did not directly measure propagule production and dispersal in this study so this effect warrants further study.

Surprisingly, bison did not have any effect on riparian woody plant cover over time. This is contrary to other studies that found bison greatly increase woody vegetation cover due to their preferential grazing on graminoid plant species thereby reducing spatial extent of burning and allowing for growth of woody plant species [5], [44]. However, bison spend little of their time within or near streams. Bison spend <6% of their time within 10 meters of streambeds at Konza Prairie and actually avoid wooded stream reaches [45]. So, although they may have an effect on woody expansion across a watershed, they likely do not within riparian zones.

Our data suggest that under current management conditions on site, woody riparian vegetation will potentially continue to expand into riparian zones, regardless of burning regimes implemented. Anthropogenic impacts on the environment, such as increased atmospheric carbon dioxide concentrations, may erase the physiological advantages that C_4_ grasses have [46]. Conversely, other studies suggest that overgrazing may be more responsible for savannahs conversion to woodlands than CO_2_ effects [47]. Our data suggest that overgrazing is not strongly related to riparian, woody encroachment therefore other abiotic factors altering ecosystem states are likely to influence woody plant species increase in riparian cover.

### Temporal variability of water yield

We did not detect any change in water yield over time for any watersheds (Fig. 4). The lowest water yield did occur in the watershed with the greatest average percentage of riparian cover. Other characteristics varied across these watersheds (number of burns, watershed area, total stream length) so we could not statistically assign the low water yield specifically to degree of riparian vegetation cover. In this study, water yield represented the proportion of stream discharge to precipitation, but one of the processes responsible for vegetation effects on water yield is evapotranspiration [48], [49], which we did not measure directly. Further, vegetation alterations can have a greater impact on the distribution of low flow periods instead of seasonal or annual water yield estimates [50]. Shrub species, such as the dominant *C. drummondii* and *Rhus glabra*, use deeper soil water sources than C_4_ grass species [15], [28] therefore replacement of riparian grasses by wooded species may reduce streamflow due to deeper roots accessibility to the water table. Headwaters at Konza Prairie have become dominated by oak species and honey locust within riparian zones [30] and their large rooting systems could be withdrawing water sources and causing reductions in discharge as well, but we have not tested water source with direct isotopic methods for these species. Conversely, forestation is known to improve infiltration capacity of soils thereby potentially offsetting streamflow reductions from greater rooting depth [50]. The antagonistic effects of water table reduction and increasing soil infiltration on hydrology may have prevented any detection of change in water yield over time.

### Conclusion

To our knowledge, this is one of the first long-term experimental manipulations of fire at the watershed level to assess the expansion rates of woody, riparian vegetation in a grassland ecosystem. We found that riparian woody vegetation cover is rapidly increasing at Konza Prairie, and although high fire frequency may slow this process, it does not necessarily cease it from occurring. This study indicates that grassy riparian corridors will be maintained only with a minimum of 2 years between burns in tallgrass prairies. We suspect that similar relationships will occur in other grasslands and fire frequency may control riparian, woody expansion though the exact relationships we found may not hold. Although we could not detect any influence of this landscape alteration on stream water yield, the level of variance may make the effect non-detectable. Long-term data collections are mandatory to effectively link land use modification to stream ecosystem dynamics so perhaps additional hydrologic collections will clarify the relationship of woody expansion to prairie stream hydrology. Conservation and management of grassland streams across the globe may require similar considerations in cases where the native condition is grass-lined stream channels.
