# Correction: Fire and Grazing Influences on Rates of Riparian Woody Plant Expansion along Grassland Streams

**DOI:** 10.1371/journal.pone.0129409

**Published:** 2015-05-26

**Authors:** Allison M. Veach, Walter K. Dodds, Adam Skibbe

The authors have found errors in this paper. These errors do not change the overall conclusions of the study.

There are errors in the last two sentences of the second paragraph of the “Spatial analysis of riparian vegetation” subsection of the Methods. The correct sentences are: Due to changes in fire frequency and other management treatments over time or the confounding effect of multiple wild fires and partially burned watersheds, only data for 20 out of 54 watersheds were retained for further analyses. Of these 20 watersheds, half were grazed, but only 1 was grazed by cattle so no differences between native and cattle grazed watersheds were determined in this study.

There are errors in Fig 1, “Spatial extent of woody plant species within a 30 m riparian buffer across the 4 watersheds of the Kings Creek basin monitored for stream discharge during 1985, 1991, and 2010.” The Natural Trail, N4A, and C1A are no longer included in the analysis. Please see the corrected Fig 1 and its legend here.

**Fig 1:**
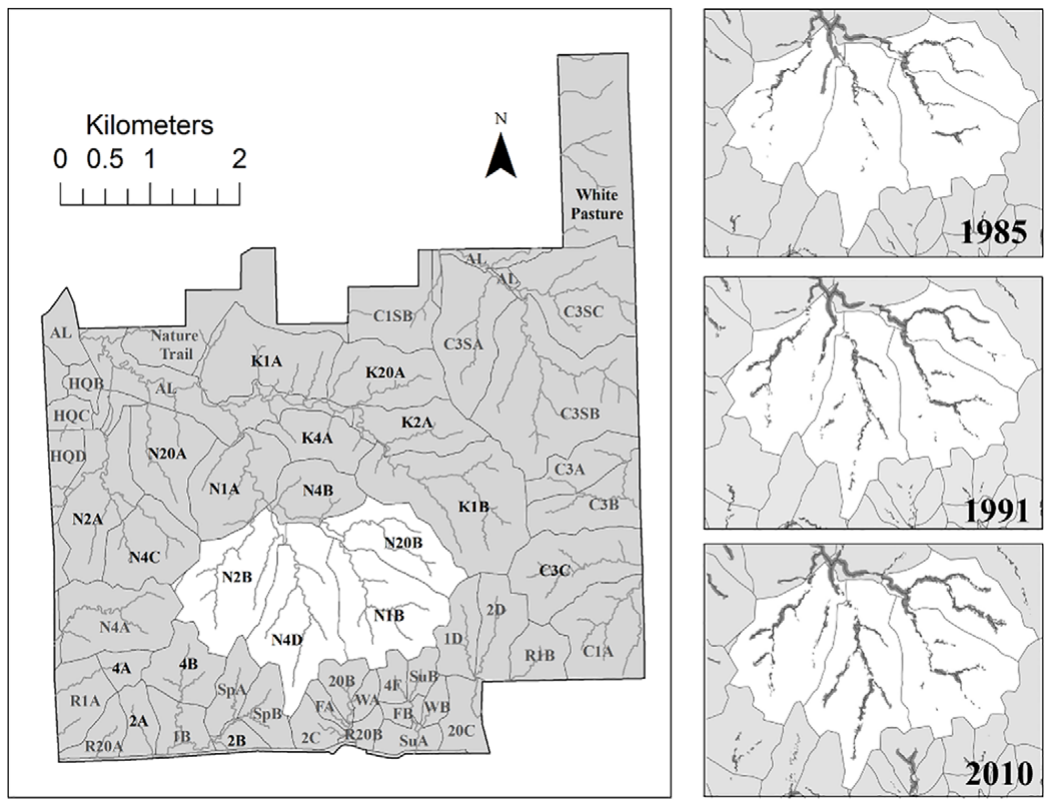
Spatial extent of woody plant species within a 30 m riparian buffer across the 4 watersheds of the Kings Creek basin monitored for stream discharge during 1985, 1991, and 2010. Woody vegetation cover within a 30 m buffer riparian zone is highlighted in gray for all three years for the 4 watersheds monitored. The 20 watersheds included in the analysis are labeled with black text whereas those not included are labeled in gray.

There are errors in the last sentence of the fourth paragraph of the “Spatial analysis of riparian vegetation” subsection of the Methods. The correct sentence is: The cumulative number of burns that had taken place between 1980 and 2010 for each watershed was collected through the Konza Prairie Biological Station LTER network burn history database [34].

There are errors in the first sentence of the “Riparian vegetation spatial analysis” subsection of the Results. The correct sentence is: Analyses of 30 m riparian buffers revealed an increase in wooded vegetation over time among all watersheds except two (Watershed 2B, β = −0.06 and White Pasture β = −0.008).

There are errors in the first sentence of the second paragraph of the “Riparian vegetation spatial analysis” subsection of the Results. The correct sentence is: Linear regression models indicated that the cumulative number of burns between 1980 and 2010, and the historical presence of woody vegetation, significantly predicted the rate of riparian vegetation expansion (P < 0.01, Adj. R^2^ = 0.51, F_3,16_ = 7.60; Fig 2).

**Fig 2:**
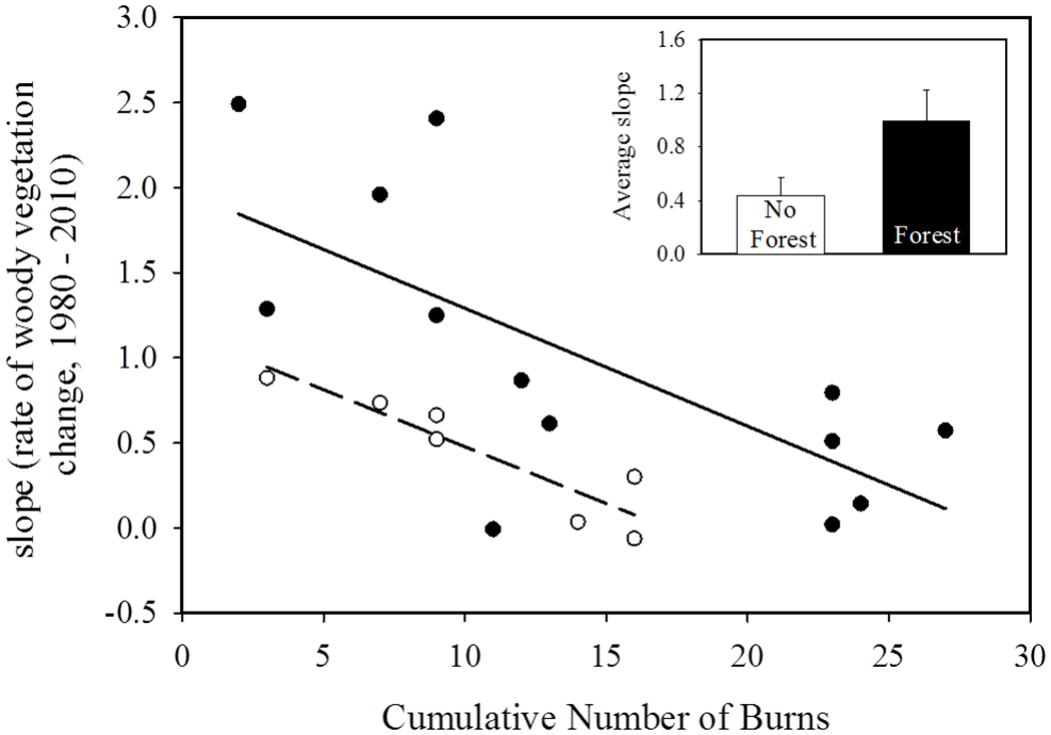
The association between the linear regression slopes calculated for each watershed’s change in riparian vegetation from 1985–2010 and the cumulative number of burns since 1980 using a multiple, linear regression model. Separate regression lines are present for watersheds without riparian woody vegetation present (open circles, dotted line), and watersheds with riparian, woody vegetation present historically (closed circles, bold line). The average slope of watersheds (rate of woody riparian expansion) with riparian forest was greater than those without forest historically (upper right panel).

There are errors in the second sentence of the second paragraph of the “Riparian vegetation spatial analysis” subsection of the Results. The correct sentence is: Further, the average rate of expansion of watersheds with forest present historically was significantly greater than those without forest (P = 0.06, T = −2.04, df = 17; Fig 2).

There are errors in the first sentence of the third paragraph of the “Riparian vegetation spatial analysis” subsection of the Results. The correct sentence is: A breakpoint was detected between burn frequency and woody expansion rate at 13 (±9.12 S.E) burns over the 30 years or at about 2.3 years between burns (Overall model: Adj. R^2^ = 0.37).

There are errors in the second sentence of the third paragraph of the “Riparian vegetation spatial analysis” subsection of the Results. The correct sentence is: Only the regression model fit on the side of the breakpoint with fewer than 13 burns had a significant slope, indicating that the cumulative number of burns significantly predicts the rate of woody expansion (P = 0.01, T = -4.18, Fig 3).

**Fig 3:**
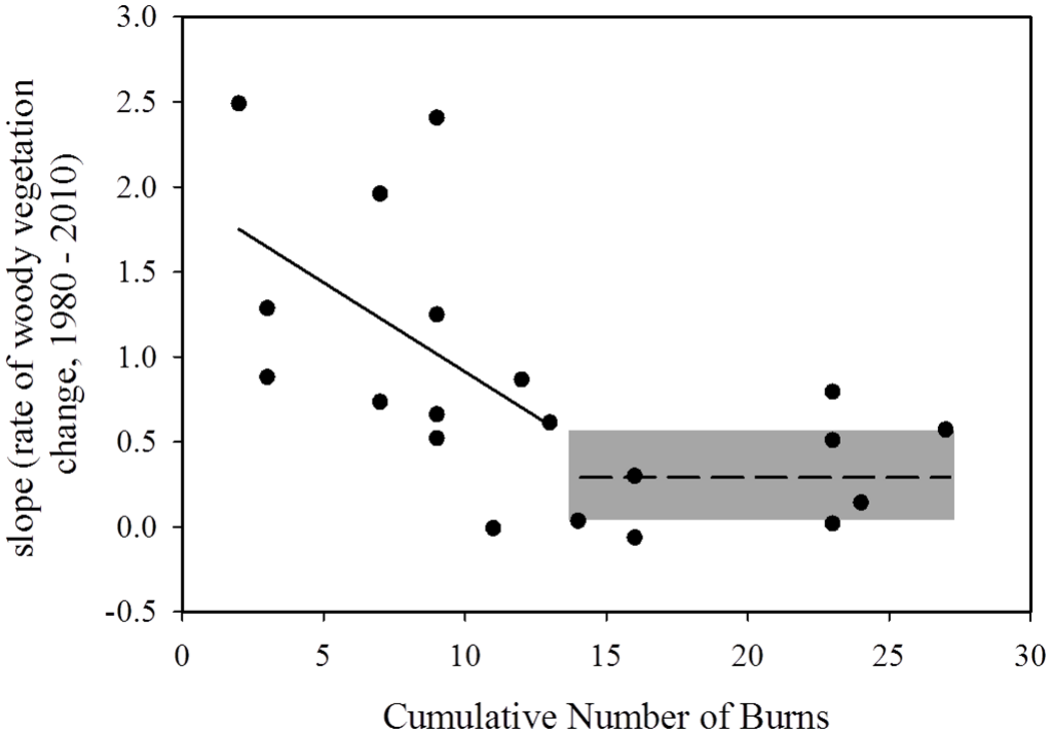
The association between the linear regression slopes calculated for each watershed’s change in riparian vegetation from 1985–2010 and the cumulative number of burns since 1980 using a segmented regression model. A breakpoint was detected at ~13 burns. The bold line represents the linear regression line for the significant portion of the regression model (watersheds with burns <13 over the 30 year record). A dashed line represents the mean of the slope of watersheds with cumulative burns >13. Gray box represents 95% confidence bands about the mean value for watersheds burned more frequently than every 2.3 years.

There are errors in the third sentence of the third paragraph of the “Riparian vegetation spatial analysis” subsection of the Results. The correct sentence is: Due to the low number of watersheds which had >13 burns over the study period, the segmented regression did not indicate a significant regression slope after the break.

There are errors in the last sentence of the third paragraph of the “Riparian vegetation spatial analysis” subsection of the Results. The correct sentence is: The rate of woody expansion for watersheds with a cumulative number of burns greater than 13 were significantly different from zero (Mean = 0.29, P = 0.03, T = 2.65, df = 7; Fig 3) indicating that burning regimes implemented more frequently than every 2.3 years may not necessarily prevent woody encroachment.

There are errors in Fig 2, “The association between the linear regression slopes calculated for each watershed’s change in riparian vegetation from 1985–2010 and the cumulative number of burns since 1980 using a multiple, linear regression model”. Please see the corrected Fig 2 and its legend here.

There are errors in Fig 3, “The association between the linear regression slopes calculated for each watershed’s change in riparian vegetation from 1985–2010 and the cumulative number of burns since 1980 using a segmented regression model”, and its legend. Please see the corrected Fig 3 and its legend here.

There are errors in the third sentence of the first paragraph of the “Factors influencing riparian, woody vegetation expansion” subsection of the Discussion. The correct sentence is: The rate of riparian woody vegetation expansion was significantly predicted by the cumulative number of burns taken place between 1980 and 2010.

There are errors in the second sentence of the second paragraph of the “Factors influencing riparian, woody vegetation expansion” subsection of the Discussion. The correct sentence is: Segmented regression results suggests that a threshold may be reached for woody vegetation cover at ~13 burns over the 30 year study period signifying that there is a change in the way riparian woody vegetation cover responds to fire when implemented every ~2 years (Fig 3).

## References

[1] Veach AM, Dodds WK, Skibbe A (2014) Fire and Grazing Influences on Rates of Riparian Woody Plant Expansion along Grassland Streams. PLoS ONE 9(9): e106922 doi: 10.1371/journal.pone.0106922 25192194 10.1371/journal.pone.0106922 PMC4156405

